# Low Cancer Incidence in Naked Mole-Rats May Be Related to Their Inability to Express the Warburg Effect

**DOI:** 10.3389/fphys.2022.859820

**Published:** 2022-05-04

**Authors:** Pedro Freire Jorge, Matthew L. Goodwin, Maurits H. Renes, Maarten W. Nijsten, Matthew Pamenter

**Affiliations:** ^1^ Department of Critical Care, University Medical Center Groningen, University of Groningen, Groningen, Netherlands; ^2^ Department of Radiology, Isala Hospital, Zwolle, Netherlands; ^3^ Department of Orthopedic Surgery, School of Medicine, Washington University St. Louis, St. Louis, MO, United States; ^4^ Department of Biology, Faculty of Science, University of Ottawa, Ottawa, ON, Canada; ^5^ Brain and Mind Research Institute, Faculty of Medicine, University of Ottawa, Ottawa, ON, Canada

**Keywords:** metabolism, Warburg effect, naked mole-rat, cancer metabolism, metabolic fuel switching, hypoxic metabolic response, hypoxic ventilatory response (HVR), thermoregulation

## Abstract

Metabolic flexibility in mammals enables stressed tissues to generate additional ATP by converting large amounts of glucose into lactic acid; however, this process can cause transient local or systemic acidosis. Certain mammals are adapted to extreme environments and are capable of enhanced metabolic flexibility as a specialized adaptation to challenging habitat niches. For example, naked mole-rats (NMRs) are a fossorial and hypoxia-tolerant mammal whose metabolic responses to environmental stressors markedly differ from most other mammals. When exposed to hypoxia, NMRs exhibit robust hypometabolism but develop minimal acidosis. Furthermore, and despite a very long lifespan relative to other rodents, NMRs have a remarkably low cancer incidence. Most advanced cancers in mammals display increased production of lactic acid from glucose, irrespective of oxygen availability. This hallmark of cancer is known as the Warburg effect (WE). Most malignancies acquire this metabolic phenotype during their somatic evolution, as the WE benefits tumor growth in several ways. We propose that the peculiar metabolism of the NMR makes development of the WE inherently difficult, which might contribute to the extraordinarily low cancer rate in NMRs. Such an adaptation of NMRs to their subterranean environment may have been facilitated by modified biochemical responses with a stronger inhibition of the production of CO_2_ and lactic acid by a decreased extracellular pH. Since this pH-inhibition could be deeply hard-wired in their metabolic make-up, it may be difficult for malignant cells in NMRs to acquire the WE-phenotype that facilitates cancer growth in other mammals. In the present commentary, we discuss this idea and propose experimental tests of our hypothesis.

## Introduction

In this commentary we address the interplay between glycolysis and oxidative phosphorylation (OxPhos) in humans and most adult mammals, with a particular focus on the physiological flexibility that is enabled by extensive exchange of lactic acid. Of particular interest is the metabolism of naked mole-rats (NMRs; *Heterocephalus glaber*), in which experimental data and metabolic logic both suggest that lactic acidosis occurs only to a very limited extent. NMRs also have a very low incidence of cancer, a disease in which extracellular lactic acidosis in the tumor microenvironment is a common hallmark.

Based on these observations, we hypothesize that the low incidence of cancer in the NMR is related to two overlapping propositions: (1) lactic acid production by NMR tissues is strongly inhibited by extracellular acidosis, and (2) this sensitivity of NMR cells to acidosis is retained by cancerous cells after malignant transformation. As a result, the Warburg effect (WE), which is central to malignant cancer progression and underlies excessive lactic acidosis in tumors, may be strongly inhibited in this species.

## Background

### Mammalian Glycolysis, OxPhos and Lactic Acid at the Cellular Level

**FIGURE 1:**
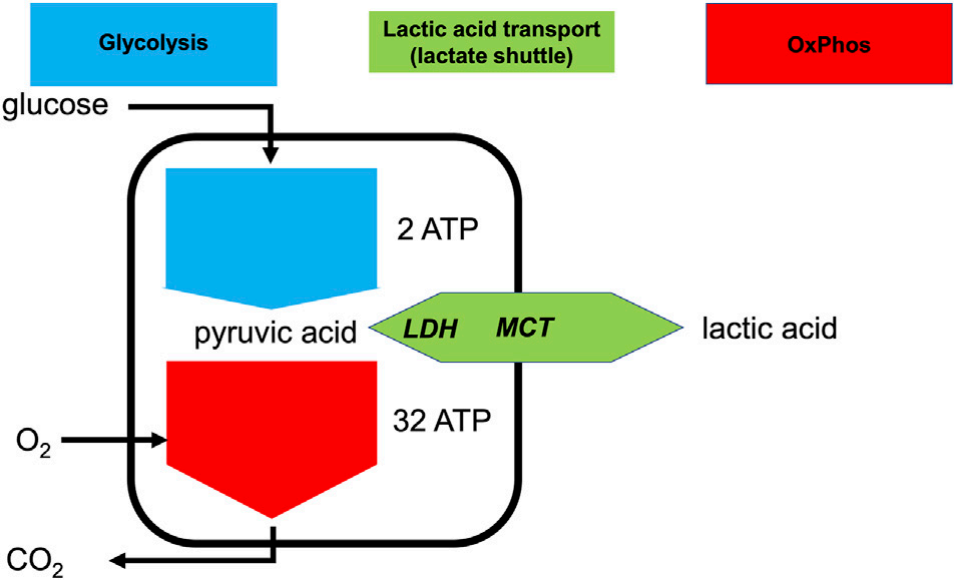
Lactate shuttling allows the uncoupling of glycolysis and OxPhos at the cellular level. Simplified depiction of aerobic and anaerobic metabolism of glucose consumption and production or consumption of lactic acid. Intracellular lactate dehydrogenase (LDH) can interconvert pyruvate and lactate and the monocarboxylate transporter (MCT) can export or import lactic acid.

Glycolysis is a ubiquitous metabolic pathway and its constituent enzymes are relatively conserved throughout evolution (Fothergill-Gilmore and Michels, 1993; Bar-Even et al., 2012). Glycolysis yields 2 ATP and 2 pyruvate molecules per glucose molecule catabolized (Bar-Even et al., 2012) (Figure 1). Oxidative phosphorylation (OxPhos) is the mitochondrial high ATP-yield pathway that uses oxygen as the terminal electron acceptor, leading to the full oxidation of pyruvate into CO_2_ and H_2_O.

Under maximally stressed conditions, such as maximal exertion, the production of pyruvate is greatly increased. Irrespective of optimal mitochondrial function, sufficient oxygen delivery, and sufficient pO_2_ at the mitochondrial level, the rate of pyruvate production can be far greater than the ability of OxPhos to metabolize it (Gladden, 2008; Brooks, 2018). Moreover, when mitochondrial metabolism cannot keep up with increased production of pyruvate, the accumulation of pyruvate and NADH will inhibit glycolysis. To allow glycolysis to continue, it is necessary for pyruvate to be reduced into lactate (La^**−**^), with the concomitant oxidation of NADH to restore NAD^+^. This near equilibrium reaction is catalyzed by lactate dehydrogenase (LDH). Together with H^**+**^, La^**−**^ can be exported to the extracellular space via monocarboxylate transporters (MCTs) (Wang et al., 2021). Essentially, all exported La^**−**^ is later metabolized by the same or different cells via the same route in reverse, i.e., intracellular uptake mediated by MCT and conversion to pyruvate by LDH (Figure 1). Importantly, although the terms lactate and lactic acid are frequently used interchangeably, lactate is exported or imported by the MCT as H^**+**^ and La^**−**^, or lactic acid (HLa) (Halestrap, 2013). Thus, whenever the term HLa is used herein, we mean the dissociated form, because given its pKa is 3.85, virtually all HLa will be dissociated *in vivo* (Kellum and Elbers, 2009).

### Whole Body Level

**FIGURE 2:**
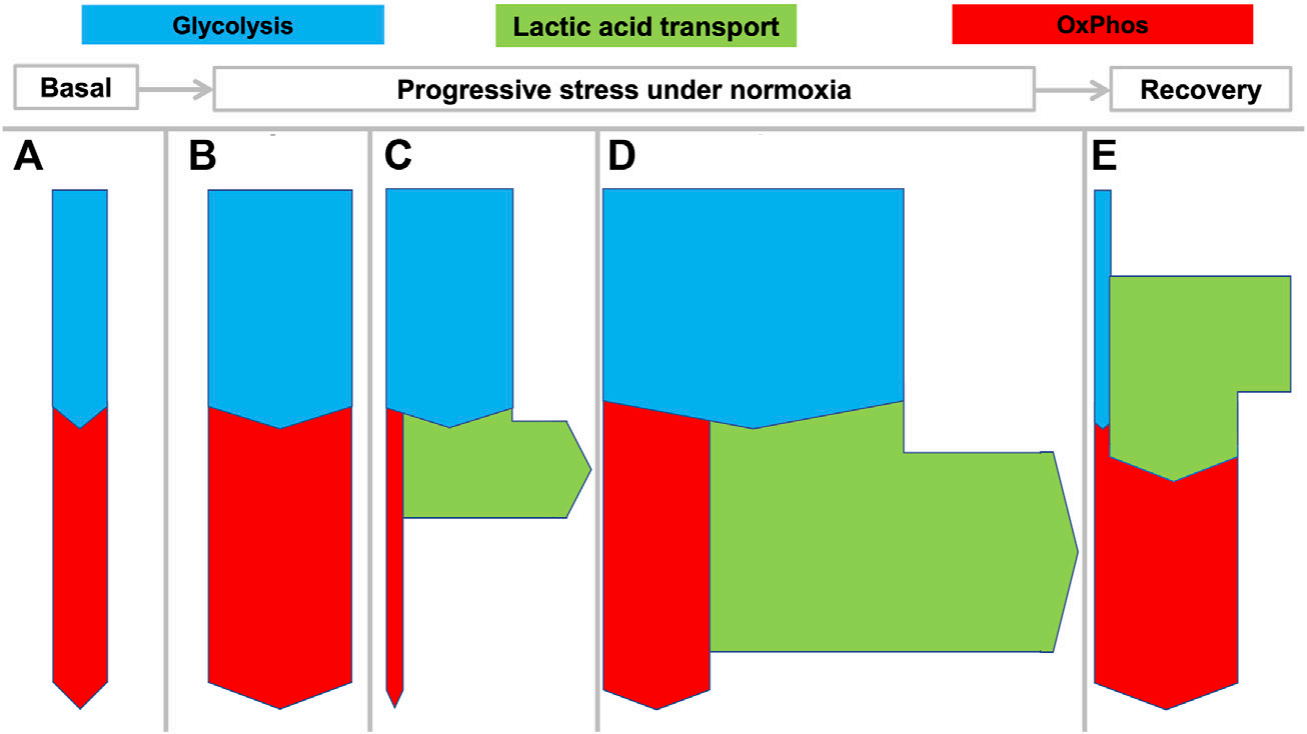
Flexible coupling of glycolysis and OxPhos in various states in mammals. Rates of net transport are indicated by the thickness of the arrows during various states as they can develop from rest to increasing stress levels and then recovery: **(A)** basal metabolism; **(B)** enhanced aerobic metabolism with matched glycolysis and OxPhos; **(C)** increased “anaerobic” glycolysis with increased lactic acid production; **(D)** increased aerobic and anaerobic metabolism as can be seen in muscle during maximal work, for example during a fight-or-flight response. “Aerobic glycolysis” is a common metabolic phenomenon that can be intermittently displayed by many tissues different from muscle to meet specific requirements. State **(E)** shows that during recovery, excess lactate can be oxidized by the same or other tissues.

Not surprisingly, LDH and MCTs are expressed in virtually all mammalian tissues (Rabinowitz and Enerbäck, 2020) and lactate is continuously produced and consumed in varying amounts by different tissues and organs. As qualitatively indicated in Figure 2, both whole body metabolism and specific tissue metabolism can display different states depending on time and the involved tissues.

Under basal conditions (Figure 2A) or mildly stressed conditions (Figure 2B) oxidation by OxPhos may match pyruvate output from glycolysis. When greater amounts of ATP are required beyond the mitochondrial capacity, glycolysis may outstrip OxPhos with the consequent production of large amounts of HLa (Figure 2C). Figure 2D illustrates even more pronounced metabolism wherein both OxPhos and lactic acid production are increased. This state is observed for example during strenuous muscle exercise wherein both oxygen and glucose consumption may be maximized.

A large percentage of the protons that enter the circulation will be buffered by bicarbonate and lead to increased CO_2_ exhalation and a decrease in bicarbonate. However, despite this buffering, the exported HLa can lead to decreases in the local or arterial pH to <6.5 and <7.0, respectively. In healthy persons, such pH levels can be observed during severe exertion but in critically ill patients they are associated with poor outcomes (Alkozai et al., 2018). Figure 2E shows how lactic acid is used as a fuel by tissue, for example during recovery after exertion.

The spatio-temporal separation of lactic acid production and consumption allows so-called lactate shuttles to function such that situations (2a) through (2e) can occur simultaneously in different tissues or sequentially in the same tissue (Gladden, 2008; Sun et al., 2017; Brooks, 2018). It is even possible for the same tissue to simultaneously produce and consume lactate (Hui et al., 2017). This gives animals and their tissues the metabolic flexibility required for many stress responses, such as the fight-or-flight reaction. Accordingly, whole-body lactate fluxes are already considerable under basal conditions (>1,000 mmol/d in humans) (Hui et al., 2017; Brooks, 2018; Rabinowitz and Enerbäck, 2020). Since only negligible amounts of lactate are taken up through food or excreted from the body, lactate is arguably the most important intermediate metabolite in mammals (Rabinowitz and Enerbäck, 2020).

**FIGURE 3:**
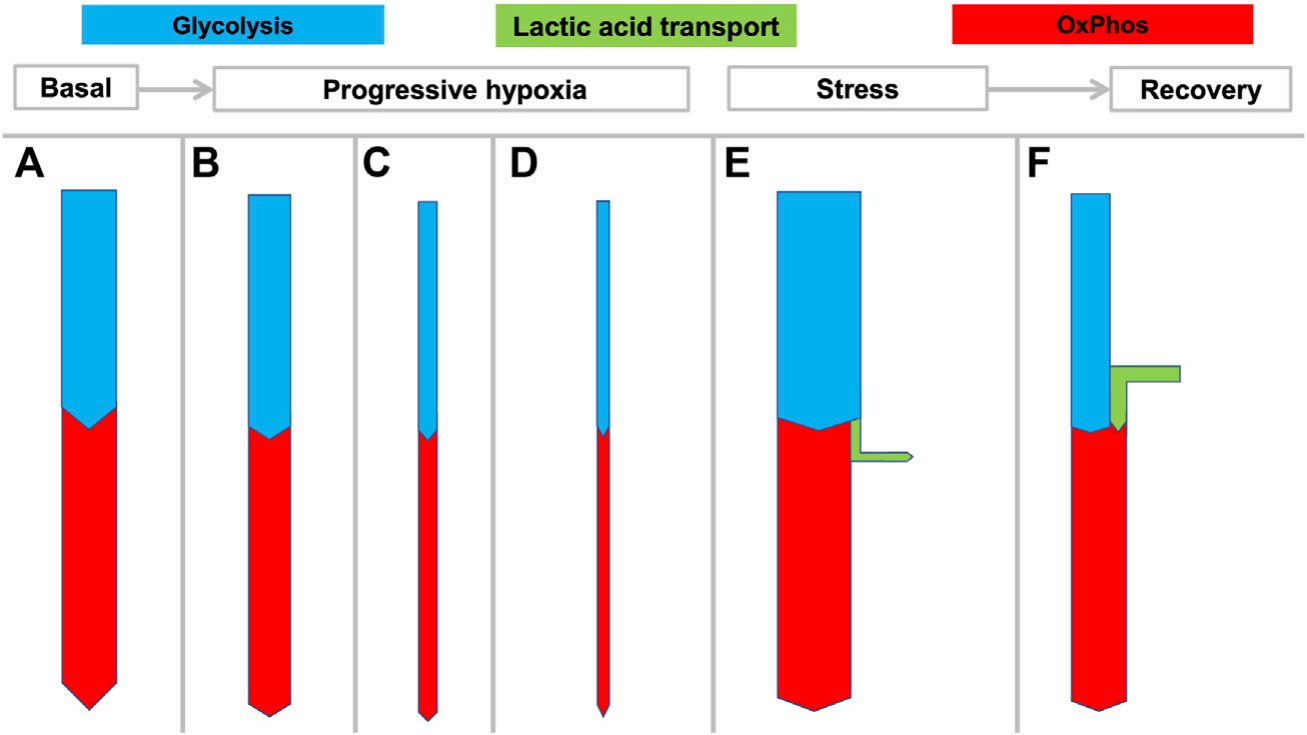
Hypothesized rigid coupling of glycolysis and OxPhos in the NMR with limited lactic acid production. States **(A–D)** reflect the large variation in oxygen consumption seen in NMRs depending on ambient temperature and oxygen availability. During hypoxia the NMR can rapidly and dramatically reduce its metabolic rate and oxygen consumption, without resorting to anaerobic glycolysis which would result in lactic acidosis. At 3% O_2_ the NMRs decreased $\dot{V}$ CO_2_ and $\dot{V}$ O_2_ by 80% and 87% respectively without developing metabolic acidosis (Pamenter et al., 2019a). State **(E)** represents the hypothesized limited lactic acid production by the NMR compared to other mammals under stress conditions since this may be inhibited by acidosis. State **(F)** depicts oxidation of excess lactate during recovery when sufficient oxygen is present.

Accordingly, Figures 2, 3 are schematic depictions of only net production or consumption of lactic acid. For the sake of simplicity, the figures do not reflect the considerably higher—but often balanced—amounts that are simultaneously being released and taken up, as it also occurs in rest.

Deep hypoxia leads to severe lactic acidosis. In humans and many mammals, a low arterial pO_2_ will generate both a systemic catecholamine-mediated stress response as well as stress at the cellular level resulting from decreasing ATP levels. Both usually induce an immediate increase of anaerobic glycolysis (Figure 2C) with increased HLa production. However, many biochemical and physiological processes can only transiently cope with severe acidosis, and a prolonged arterial pH < 7.0 is not compatible with life. Since clearance of an increasing HLa load (Figure 2E) under hypoxia is not possible, sustained progressive lactic acidosis will result until the patient or animal dies.

When successful recovery from hypoxia or severe exertion occurs, excess anaerobically generated HLa is cleared from the body. The HLa is either fully oxidized to CO_2_ (Figure 2E) or regenerated to muscle glycogen or glucose through hepatic or renal gluconeogenesis. Since both processes consume oxygen, this phase is characterized by increased oxygen consumption ( $\dot{V}$ O_2_) to restore the nominal situation, so the “oxygen debt” that developed during hypoxia is “repaid” during recovery (Gaesser and Brooks, 1984). Importantly, the states depicted in Figures 2D,E can coexist. During the Cori cycle muscles convert glucose to HLa and the liver converts it back to glucose (Cori, 1981). In this manner the liver can offload muscle metabolism, although this cycle carries a net cost of 4 ATP (National Center for Biotechnology Information, 2021) for the organism as a whole, and thus increases $\dot{V}$ O_2_ (National Center for Biotechnology Information, 2021).

### The NMR and its Metabolism

NMRs live mainly in east Africa (Jarvis and Sale, 1971), where large colonies spend virtually their whole life in extensive and sometimes poorly ventilated tunnel systems. To cope with this environment, the NMR possesses some physiological features that are strikingly different from most other adult mammals, and these features support the ability to survive severely challenging levels of hypercapnia and hypoxia. With a lower basal metabolic rate (MR) than expected for its body mass (Lovegrove, 1986), the NMR is an exception to the allometric principle which states that - after log-log transformation - basal MR correlates linearly with body mass (White and Seymour, 2003). Likewise, the NMR has lower circulating glucose levels than other similarly sized mammals (Umminger, 1975; Park et al., 2017). The NMR’s natural habitat is believed to be both hypoxic and hypercapnic in more densely-populated areas (Buffenstein et al., 2022; Pamenter, 2022). Hypercapnia can induce respiratory acidosis and hypoxia can induce metabolic (lactic) acidosis. Cellular acidosis also has significant effects on aerobic mitochondrial respiration because deviations from physiological pH (acidic or alkaline) may shift the mitochondrial H^+^ gradient, alter the kinetics of the various enzymes of the mitochondrial electron transport chain (ETC), impact reactive oxygen species (ROS) generation, or damage mitochondrial components, among other effects (Forman and Wilson, 1982; Holtzman et al., 1987; Selivanov et al., 2008; Santo-Domingo and Demaurex, 2012; Genders et al., 2019). Thus, unbuffered changes in pH may further impair ATP production in hypoxia and promote cell death.

### Hypercapnia

The NMR tolerates ambient CO_2_ levels of <7.5% without hyperventilating or developing systemic acidosis, and can survive 80% CO_2_ for 5 h, which is lethal to mice within 5 min (Park et al., 2017). An important component of this resistance is the loss of the strong sensitivity to ambient CO_2_ that other mammals possess. Since above-ground CO_2_ is 0.04% and the end-expiratory CO_2_ may be ∼5%, direct or indirect sensing of arterial CO_2_ closely controls minute ventilation in most animals, and thus hypercapnia is a key driver of hyperventilation. But apart from this important sensing consideration where NMRs possess relatively blunted hypercapnic responses (Clayson et al., 2020), high ambient CO_2_ or high arterial pCO_2_ as such do not preclude “normal” metabolism, as demonstrated by chronically hypercapnic patients with severe chronic lung disease (Rose and Post, 2001). The usual adaptation in mammals to hypercapnia is to increase circulating bicarbonate levels to restore a normal pH. The Henderson-Hasselbalch equation relates pH, pCO_2_ and the bicarbonate concentration:

$$pH=c+log_{10}\left(\left[HCO_{3}^{-}\right]/pCO_{2}\right)$$

where c depends on temperature and units used. When pH and pCO_2_ are known, the bicarbonate level is determined accordingly. Subsequently, acid-base principles in mammals (Rose and Post, 2001; Kellum and Elbers, 2009), imply that this increase in bicarbonate requires an adaptive response from the kidneys or gut that leads to an increase in the strong-ion difference. As the strong ion difference is mainly determined by the difference between [Na^+^] and [Cl^−^], this response involves either enhanced absorption of sodium and/or enhanced excretion of chloride (Rose and Post, 2001; Kellum and Elbers, 2009), both leading to increased bicarbonate. When the environmental CO_2_ level is increased, the arterial pCO_2_ must be at least equal to the environmental pCO_2_ and when the observed pH then remains (initially) stable, bicarbonate must have increased (Park et al., 2017; Fig S1). It is not yet clear how the NMR might rapidly achieve elevated bicarbonate levels.

### Hypoxia

Hypoxia poses a more fundamental challenge than hypercapnia for the NMR (and other mammals) (Storz and McClelland, 2017). Since physiological constraints offer only limited possibilities to increase oxygen transport or extraction, oxygen consumption must usually decrease in hypoxia to maintain metabolic equilibrium (Buck and Pamenter, 2006). Whereas hypoxia-intolerant mammals typically attempt to upregulate anaerobic metabolism to maintain ATP supply when oxygen is limited, most hypoxia-tolerant species instead exhibit robust metabolic rate suppression (Seagroves et al., 2001; Hochachka and Somero, 2002). This is presumably because upregulating anaerobic metabolism is limited by finite systemic glycogen stores to fuel glycolysis, and is maladaptive due to the occurrence of progressive acidosis resulting from the accumulation of acidic metabolic end-products (primarily lactate) (Gaesser and Brooks, 1984; Gladden, 2008; Sun et al., 2017). Notably, although fructose metabolism is upregulated in NMRs during anoxia (Park et al., 2017), compared to glucose this does not offer benefits in terms of ATP-production.

Nonetheless, all species must use glycolytic throughput to at least partially compensate for a drastically reduced ability to produce ATP in severe hypoxia and thus many hypoxia-tolerant species have developed elegant strategies to ameliorate the impact of lactic acidosis arising from sustained reliance on anaerobic metabolism. For example, the production of alternative end-products reduces or prevents backlogs of metabolic pathways and cellular acidification in some species (Shoubridge and Hochachka, 1980; Fagernes et al., 2017), while others have developed robust pH-buffering capacities (Jackson 1997; Jackson, 2000; Reese et al., 2004).

Under experimental hypoxia (from 9–3% O_2_), NMRs rapidly decrease their $\dot{V}$ O_2_ by up to 85% (Pamenter et al., 2018; Figure 3), thus achieving a maximally hypometabolic state (Ilacqua et al., 2017), which is a different or even opposite response from that displayed by mice or humans, in which metabolism is often unchanged or only mildly decreased in hypoxia (Hochachka and Somero, 2002). For example, small mammals such as mice can to some extent reduce their metabolism under hypoxia (Gu and June 2018), albeit not to the degree as displayed by NMRs. Many larger mammals, such as humans, cannot shift to hypoxic metabolism under such circumstances, and may develop a stress response that increases oxygen debt.

Moreover, NMRs do not develop marked systemic acidosis, which occurs in above-ground mammals during hypoxia (Hochachka and Somero, 2002). In fact, after 4 h of progressive hypoxia down to an ambient O_2_ of 3%, no acidosis but even a mild but significant rise in pH and only a minimal decrease of base excess is observed in most tissues (except in brain, see below) (Pamenter et al., 2019a). Under these conditions the respiratory quotient (RQ; = $\dot{V}$ CO_2_/ $\dot{V}$ O_2_) rises towards 1.0, pointing to a switch from lipid to carbohydrate metabolism, which is metabolically advantageous under limited oxygen supply. Furthermore, the characteristic increase in $\dot{V}$ O_2_ to repay oxygen debt is not observed in NMRs after reoxygenation. Although lactate levels have not been measured in hypoxic NMRs, these results strongly suggest that a major systemic rise in lactate is absent. In addition, expression of glucose transporter 4 (GLUT4, the primary mediator of glucose uptake into cells), glycogen synthase (a key regulatory enzyme of glycolysis), and the phosphorylated form of AMP kinase (AMPK; the upregulation of which typically mediates increased glycolysis) (Hardie et al., 2012), all decrease in NMR skeletal muscle following 4 h of *in vivo* hypoxia (Hadj-Moussa et al., 2021a), changes which are all consistent with decreased glycolytic throughput.

On the other hand, some *in vivo* evidence supports reliance on glycolytic metabolism in the hypoxic NMR, at least to some degree and in certain tissues. For example, in hypoxia we observe mobilization of liver glycogen stores and increase in blood glucose levels. The increase in blood glucose levels may also partly be explained by decreased glucose consumption during hypometabolic states (Pamenter et al., 2019a). In the brain, and following acute *in vivo* hypoxic exposure, mild acidification is observed, lactate dehydrogenase (LDH) protein expression increases, and the expression of SREP2, a key regulator of fatty acid synthesis, decreases (Hadj-Moussa et al., 2021b), all consistent with increased glycolytic throughput in this tissue. Finally, NMRs have large cardiac glycogen stores relative to C57/BL5 mice (Faulkes et al., 2019), suggesting this organ is primed for sustained glycolytic metabolism. Indeed, it is metabolically advantageous in terms of produced ATP and H^+^ for the myocardium to use glycogenolysis compared to importing circulating glucose as glycogenolysis does not require the initial ATP investment that occurs when imported glucose is phosphorylated in the first step of glycolysis (Allard et al., 1997).

Thus, the degree to which NMRs rely on glycolysis in acute hypoxia is an open-ended question and likely depends on the severity and duration of hypoxic exposure. It is clear that NMRs can generate lactate (Park et al., 2017), and the moderately elevated lactate levels in brain slices are comparable between mice and NMRs (∼3 umol/g). In rats, blood lactate levels of ∼14 mmol/L have been observed after 1 h of 10% inspiratory oxygen (Alva et al., 2010). Blood lactate levels have to our knowledge not been measured in the NMR under such conditions. However, the observed normal pH of the blood combined with the absence of hyperventilation during hypoxia in NMRs (Pamenter et al., 2019a) effectively precludes the accumulation of the amount of lactic acid seen in other mammals during hypoxia. This suggests that, even if glycolysis is important in certain tissues during hypoxia, localized lactate production in a few organs does not translate to significant increases at the organismal level. Indeed, given the overall capability of NMRs to rapidly reduce $\dot{V}$ O_2_ in hypoxia, allowing unabated HLa production would only be a very short-term solution with distinct disadvantages, including progressive acidosis and accruing an oxygen debt during hypoxia that may last for an unknown duration. Notably, while the comparatively higher hemoglobin and hematocrit observed in NMRs allows the blood to have higher non-bicarbonate buffering power, this cannot explain the remarkable lack of systemic acidification under hypoxia. Taken together, these observations strongly suggest that unlike other mammals, the NMR never develops marked systemic lactic acidosis.

### NMR Lifespan and Cancer Incidence

NMRs can live for decades, which is an order of a magnitude more than observed for other mammals with a similar body mass (White and Seymour, 2003; Healy et al., 2014). Despite their long lifespan, NMRs also have an extraordinarily low incidence of cancer compared to other animals (Buffenstein, 2005; Tian et al., 2013; Deweerdt, 2014; Healy et al., 2014; Taylor et al., 2017; Bredberg and Schmitz, 2019). Various hypotheses have been suggested to explain the low incidence of cancer in NMRs (Anonymous, 2013; Miyawaki et al., 2016; Bredberg and Schmitz, 2019). For example, high-molecular-mass hyaluronan, as expressed in the extracellular matrix of NMRs, has been proposed as a contact inhibitor of tumor growth (Tian et al., 2013), although a more recent study was unable to reproduce key observations underlying this hypothesis (Hadi et al., 2020). Alternatively, some authors have suggested that the positive relationship between animals’ longevity and body size may related to the relatively lower incidence of cancer in larger animals, since these animals have a lower specific MR (i.e. BMR indexed for body weight) (Dang 2015). NMRs also maintain lower basal levels of pleiotropic hormones including thyroxine, cortisol and insulin, which appears to be intimately connected to expanded lifespan (Buffenstein et al., 2001; Buffenstein and Pinto 2009). Intriguingly, others have noted that the ability of NMRs to withstand hypoxia may be related to both their very long lifespan and their low cancer incidence, for example because of better defenses against oxygen radical formation (Rodriguez et al., 2011; Hawkins et al., 2019; Munro et al., 2019).

### The Warburg Effect

A large majority of solid and hematological malignancies acquire the WE (Vander Heiden et al., 2009) between the initiation of abnormal growth resulting from a mutated cell and the time when a macroscopic cancer becomes clinically detectable (Warburg et al., 1927). The WE is a final common metabolic phenotype that can result from various mutations and is recognized as one of the hallmarks of cancer (Hanahan and Weinberg, 2011). The WE involves the uptake of large amounts of glucose and the export of lactic acid by tumors, irrespective of the presence of oxygen (Warburg et al., 1927). Otto Warburg claimed that these findings were caused by disruptions in mitochondrial bioenergetics (Warburg, 1956); however, this has been demonstrated not to be true and in fact many studies indicate that tumoral mitochondrial function remains intact (Borst, 1960; Racker, 1972).

The WE supports many of the tumor’s invasive purposes (Vander Heiden et al., 2009). The WE boosts biomass generation of the tumor (Vander Heiden et al., 2009) but also results in considerable release of lactic acid to the extracellular space (Horsman and Vaupel, 2016; de la Cruz-Lopéz et al., 2019; Ji et al., 2019; Boedtkjer and Pedersen, 2020). This local acidosis also assists tumor growth as it can degrade the tumor environment and is associated with increased resistance to chemo-, radio- and immuno-therapy (Roma-Rodrigues et al., 2019; Boedtkjer and Pedersen, 2020). Importantly from a clinical perspective, this large glucose uptake by cancers that display the WE is the basis of the extra-ordinary utility of the glucose analogue fluorodeoxyglucose positron emission tomography (FDG-PET) for diagnosis and follow-up of many cancers (Hustinx et al., 2002; Mandelkern and Raines, 2002; Ide and Suzuki, 2005).

It should be underscored that the so-called aerobic glycolysis, i.e., the conversion of glucose to lactic acid while abundant oxygen is available, occurs commonly in physiology, so it is not unique to the WE. Aerobic glycolysis is a common metabolic phenomenon that can be intermittently displayed by many tissues to meet specific requirements, e.g., by the muscles during a sprint (Figure 2D). The key distinction between the WE and physiological aerobic glycolysis, is that the WE is a continuous process that is not under physiological control.

## Hypothesis

**FIGURE 4:**
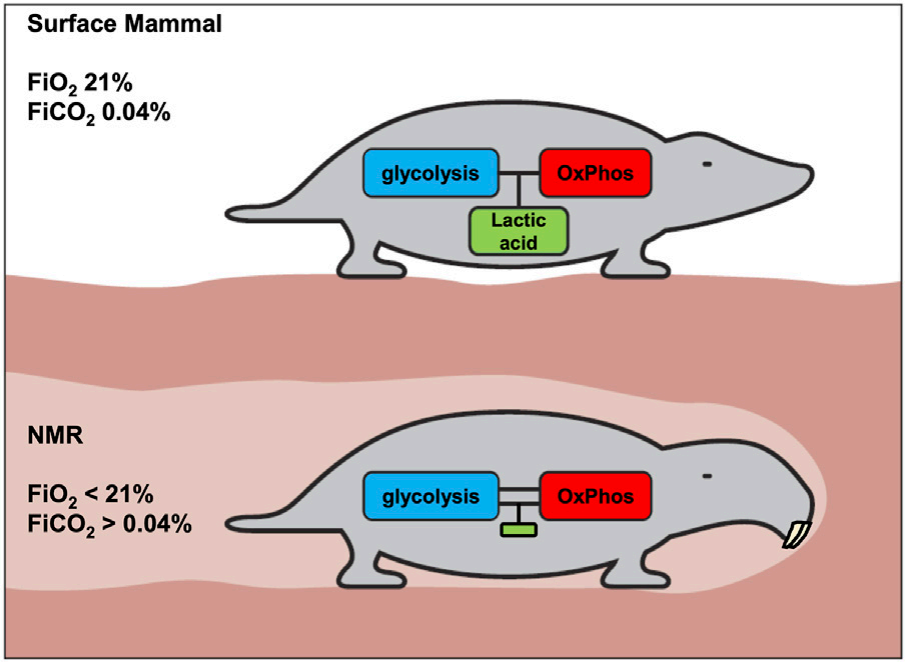
Proposed metabolic difference between surface mammals and NMRs with respect to coupling of glycolysis and OxPhos. The generation of lactic acid allows tissues to more flexibly generate ATP. A consequence of this flexibility is transient regional or systemic acidosis. We hypothesize that acidosis is strongly inhibited in the NMR, and that consequently the coupling of glycolysis and OxPhos must be much stronger in NMRs, to avoid undesired pH-decreases. Accordingly, it is proposed that, compared to above-ground mammals, the NMR will display less acidosis and lower lactate levels under various stress conditions.

Based on the principles and data discussed above, we conclude that unlike for other mammals (Figure 4, upper panel), uncoupling of glycolysis and OxPhos does not confer advantages to the NMR, which has devised a unique strategy of immediate hypometabolism when confronted with hypoxia.

Therefore, we hypothesize that:

a) in the NMR (or in most NMR tissues), glycolysis and OxPhos are strongly coupled (Figure 4, lower panel) to avoid either systemic or local lactic acidosis resulting from glycolysis outrunning OxPhos. Strong inhibition of glycolysis by lowered pH would be the most obvious mechanism for the NMR to achieve this, given pH’s central role in regulating biochemical reactions.
b) since it is important that this inhibition should hold across tissues and organs, the higher pH-setpoint for inhibition of glycolysis could be deeply hardwired in the NMR’s biochemistry. Thus, even malignant cells cannot circumvent this inhibition to develop the WE.

It should be noted that the observation in NMRs of mildly elevated lactate levels without acidosis, as has been reported under normal FiO_2_ conditions (Holtze et al., 2020) does not contradict this hypothesis. Lactate can be produced by stressed tissues and consumed when sufficient oxygen is available, as long as acidosis is avoided. But in contrast to above-ground mammals that can physiologically develop marked hyperlactatemia and acidosis (i.e., lactate>10 mmol/L and arterial pH < 7.0), this may not occur in the NMR.

Notably, metabolic demand may overcome this limitation in certain tissues in which glycolysis may proceed to some extent (e.g., brain) but NMRs appear to possess numerous defenses against cancer progression, which may explain low incidence rates of brain cancer in this organism, even if the WE were not inhibited in this tissue.

## Testing the Hypothesis

Several clear implications of the strong inhibition of glycolysis by acidosis could be verified through comparisons of NMRs with other animals.

At the whole animal level, the most straightforward test would be to perform blood gas analyses, including lactate measurements, in NMRs under hypoxia. Although hyperlactatemia has been abundantly demonstrated in many mammalian models, this has unfortunately not been assessed in NMRs, since *in vivo* blood sampling is far more difficult in the NMR than in mice due to anatomical differences. Once this technical challenge is addressed, our hypothesis predicts lower lactate levels in NMRs than in mice under hypoxia and other comparable stress conditions such as heavy exertion. Alternatively, artificially induced metabolic acidosis (e.g., through oral administration of ammonium chloride) should decrease glycolysis and induce hypometabolism. Likewise, administration of lactate to NMRs in the form of HLa or as sodium lactate (NaLa) could be used to compare the ability of NMRs to clear lactate with that of other animals. Labelling with stable isotopes of important substrates may help define how metabolic fluxes differ and how they are affected by pH changes.

Alternatively, at the tissue or cellular levels, the coupling of glycolysis and OxPhos could be compared *in vitro* under various conditions, including under varying oxygen, glucose and lactate concentrations, and for different extracellular pH levels, all of which are relatively easy to manipulate in these *ex vivo* systems. It would also be instructive to determine the pH sensitivity of the enzymes of glycolysis (e.g., phosphofructokinase) in the NMR. Likewise, the general expression of LDH or MCT’s may be lower, leading to lower generation of HLa. Alternatively, post-translational changes in LDH might result in lower enzyme activity (Pamenter et al., 2019b).

Beyond these “baseline” measures, several cancer-targeted investigations could help test our hypothesis. Only a few cases of cancer in NMRs have been reported and described in detail postmortem (Taylor et al., 2017). Samples from these cancers, if obtained, could support histochemical and molecular analysis to establish if they do or do not conform to the Warburg phenotype. Alternatively, xenografts of modified NMR cells transplanted into mice have been performed (Tian et al., 2013), and these tumors could be assessed for the WE, for example with FDG-PET scanning. In the case of xenografts when increased tissue glycolysis is observed, it should be verified if host cells such as fibroblasts or the malignant cells are contributing to the WE. Cultured malignant transformed NMR cells could also be assessed to determine whether or not they have a strongly glycolytic profile as seen for many cancer cells from other animals. Likewise, the effect of the pH of the culture medium on growth and metabolism could be examined.

## Conclusion

It is not unreasonable to assume that the NMR’s unique metabolism, which suits its underground habitat, is in some way related to its remarkable resistance to cancer. The ability of NMRs to tolerate and exercise in hypoxic environments without significant acidosis is a striking metabolic characteristic of this mammal. Thus, we hypothesize that the low cancer incidence in the NMR may be related to a hard-wired coupling of glycolysis with OxPhos leading to the inability to express the Warburg effect, a phenotype that characterizes most cancers in other mammals.

## Abbreviations

ATP, adenosine triphosphate; BMR, basal metabolic rate; FDG-PET, fluorodeoxyglucose positron emission tomography; HLa, lactic acid; La^−^, lactate; LDH, lactate dehydrogenase; MCT, monocarboxylic acid transporter; MR, metabolic rate; NADH, Nicotinamide-adenine-dinucleotide; NMR, naked mole-rat; OxPhos, oxidative phosphorylation; pCO_2_, pO_2_; RQ, respiratory quotient; $\dot{V}$ CO_2_, CO_2_-production; $\dot{V}$ O_2_, O_2_-consumption; WE, Warburg effect.
